# Signal Intrusion Explains Divergent Effects of Visual Distraction on Working Memory

**DOI:** 10.1177/09567976251331039

**Published:** 2025-05-05

**Authors:** Ziyao Zhang, Jarrod A. Lewis-Peacock

**Affiliations:** Department of Psychology, the University of Texas at Austin

**Keywords:** working memory, memory distortion, distraction interference

## Abstract

Perceptual distraction distorts visual working memories. Recent research has shown divergent effects of distraction on memory performance, including attractive biases, impairment of memory precision, and an increase in the guess rate, indicating multiple mechanisms of distraction interference. Here we propose a novel signal-intrusion model based on the TCC (target-confusability-competition) framework to reconcile those discrepant results. We hypothesized that sensory interference is driven by the integration of a target signal and an intrusive distractor signal. Model comparisons showed that this TCC-intrusion model had a superior fit to memory error distributions across three delayed-estimation tasks with distraction (*N* = 220 adults) compared with other candidate models. According to the model, distractor intrusions decreased along with target-distractor dissimilarity, in accordance with the sensory-recruitment hypothesis. Moreover, TCC-intrusion successfully replicated divergent effects of distraction on memory bias, precision, and guess rate using this one intrusion mechanism. Together, these results suggest that perceptual distractors affect working memories through a unified mechanism of signal intrusion.

## Introduction

Memories are not fixed entities but are instead the result of a reconstructive process that is adaptive and labile. The act of retrieving episodic memories can place them in a modifiable state, rendering them susceptible to integration with new experiences—a phenomenon known as *reconsolidation* (Lee et al., 2017; Walker et al., 2003). In certain instances, the reactivation of old fear memories can lead to their intermingling with new extinction experiences, thereby preventing the resurgence of fear responses (Schiller et al., 2010). Similarly, visual working memories are not static entities. Active retention of visual memories can be distorted by the processing of external visual information, a phenomenon referred to as the *sensory-interference effect* (Clapp et al., 2010; Teng & Kravitz, 2019; for a comprehensive review, see Lorenc et al., 2021).

The sensory-interference effect has been replicated consistently across various working memory paradigms, yet the underlying mechanisms remain elusive. In recognition tasks, the inclusion of perceptual distractors during the maintenance phase has been found to impair task accuracy (Allen & Ueno, 2018; Bloemendaal et al., 2015; Clapp & Gazzaley, 2012; Clapp et al., 2010; Feredoes et al., 2011; Hermann et al., 2021; Hitch et al., 2018; Hu et al., 2014; Jha & Kiyonaga, 2010; Jha et al., 2004; Yoon et al., 2006; but see Bettencourt & Xu, 2016). The detrimental effect of distractors is modulated by the similarity between targets and distractors. Memory performance is more severely affected when there is greater feature overlap between the distractor and the memory target (Bloemendaal et al., 2015; Hermann et al., 2021; Jha & Kiyonaga, 2010; Jha et al., 2004; Yoon et al., 2006). For example, memory accuracy for faces is more impaired by face distractors than by scene distractors (Yoon et al., 2006). Similarly, for low-level features such as color and shape, distractors subjectively similar to maintained working memories induce larger attractive biases compared to subjectively dissimilar distractors (Fukuda et al., 2022; Saito et al., 2020).

These findings of perceptual interference and congruency effects align with the sensory-recruitment account of visual working memory. This account posits that visual working memory and visual perception partially overlap in neural substrate and coding formats (D’Esposito, 2007; D’Esposito & Postle, 2015; Harrison & Tong, 2009; Postle, 2006; Serences et al., 2009). Processing visual information while maintaining visual memories induces interference between their neural codes. This interference becomes more pronounced as the overlap between perceived information and maintained representation becomes more similar (D’Esposito, 2007; D’Esposito & Postle, 2015; Scimeca et al., 2018).

**Fig. 1.**
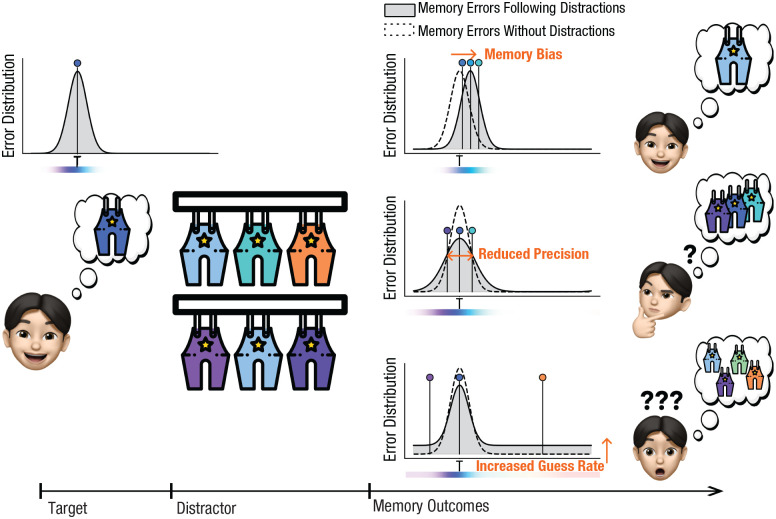
Illustrations of varied distraction costs found in the literature. Distractions have been shown to result in several types of memory-related costs, including memory bias, reduced precision, and increased guess rates. Memory bias manifests as memory errors that shift toward distractor information. Reduced memory precision is indicated by an increase in the standard deviation of error distributions, reflecting greater variability in memory responses. An increased guess rate is reflected by the heightened tails of the error distribution, suggesting a higher proportion of random responses. Gray-filled distribution plots represent the error distributions following distractions. Dashed distribution plots represent the error distributions without distractions. Three-point estimates are sampled from each error distribution to illustrate possible memory outcomes. T indicates target information and thus a error of zero degree.

Recent advances in mixture modeling and delayed-estimation paradigms have revealed various types of memory errors induced by perceptual distractions, including memory bias, reduced memory precision, and increased guess rates. In delayed-estimation paradigms, participants are asked to reproduce a memorized feature (e.g., by selecting a color on a continuous response wheel representing all possible values). Errors are measured as the distance between the chosen feature and the target feature, with most errors centered around zero, forming a Gaussian-like distribution; occasional larger errors appear at the edges of this central distribution (see Fig. 1). Mixture models have been developed to analyze these error distributions, estimating (a) the proportion of responses likely driven by accurate target memory, represented by the central Gaussian distribution; (b) memory precision, reflecting the spread of the Gaussian distribution; and (c) the proportion of guess responses, indicated by the height of the flat tail component, as random guesses are uniformly distributed across the feature space (Zhang & Luck, 2008).

A modified version of the mixture model has been adapted to assess distraction effects on memory performance by allowing the central Gaussian distribution to shift, capturing memory bias toward distractors. Such an attractive bias results in error clustering around distractor information (Fukuda et al., 2022; Gresch et al., 2021; Hallenbeck et al., 2021; Lorenc et al., 2018; Mallett et al., 2020; Rademaker et al., 2015; Saito et al., 2020; Teng & Kravitz, 2019; Van der Stigchel et al., 2007; Zhang & Lewis-Peacock, 2022, 2023; for a review, see Lorenc et al., 2021).

Distractions affect not only memory bias but also memory precision and guess rate in mixture models (see Fig. 1). Memory precision reflects the spread of memory errors, with reduced precision indicated by increased error-distribution variance, suggesting greater variability in memory responses (Li et al., 2020; Rademaker et al., 2015; Sun et al., 2017; Teng & Kravitz, 2019). The guess rate, likely because of random guesses, contributes to a uniform error distribution and appears as heightened tails in the error distribution following distractions (Li et al., 2020; Sun et al., 2017).

These varied distraction costs showed a complex relationship with target-distractor distance, directly contradicting the predictions of the sensory-recruitment hypothesis. Biases induced by perceptual distractors follow a nonmonotonic similarity function. Specifically, biases increase initially as the target-distractor distance in the feature space (dissimilarity) increases, but then biases decrease as the dissimilarity increases further (exhibiting an inverted-U-shape pattern; Gresch et al., 2021; Nemes et al., 2011, 2012; but see Mallett et al., 2020; Rademaker et al., 2015). This conflicts with the monotonic congruency effects of distractions observed in recognition paradigms, diverging from predictions by the sensory-recruitment hypothesis. This disparity between recognition and estimation paradigms stands as a crucial gap in the literature.

The impact of distractors on memory precision and guess rate, as estimated by mixture modeling, also directly contradicts the sensory-recruitment hypothesis and suggests multiple dissociable mechanisms of distraction effects (see Fig. 1). In terms of memory precision, prior research has demonstrated that sensory distractors similar to the target (2° and 7° in the orientation space) resulted in increased memory precision (Rademaker et al., 2015). Conversely, sensory distractors less similar to the target (60° and 90° in the color space, 45° in the orientation space) led to reduced memory precision (Rademaker et al., 2015; Sun et al., 2017). Regarding guess responses, similar distractors (30° and 60° in the color space, 15°–75° in the shape space) decreased guess rates (Li et al., 2020; Sun et al., 2017), whereas dissimilar distractors (90°, 120°, and 150° in the color space, 105°–165° in the shape space) increased guess rates (Li et al., 2020; Sun et al., 2017). In summary, using mixture modeling to investigate sensory-interference effects in prior research revealed that distractors similar to the target induce attractive biases, with potential effects of increasing memory precision and reducing guess rates. Dissimilar distractors, although less likely to induce response biases, may produce undesirable effects of decreased memory precision and increased guess rates. The divergent effects of sensory interference on biases, memory precision, and guess rates cannot be explained by the sensory-recruitment hypothesis. These contradictory findings instead suggest that sensory interference may be driven by different mechanisms in different contexts.

**Fig. 2.**
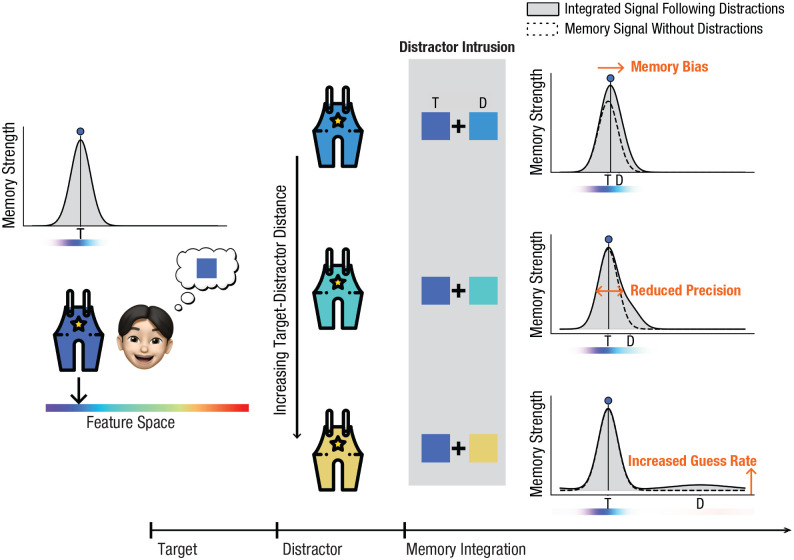
Illustrations of how signal intrusion explains divergent distraction costs. Dashed lines represent pure target signals; solid lines represent the final signal that was produced by the integration of the target and the distractor signals. The integration of these signals can account for varied empirical phenomena (target-distractor distance increases from top to bottom). Distractors that are similar to the target induce attractive biases but enhance precision. Distractors that are moderately similar to the target reduce memory precision, whereas highly dissimilar distractors increase the guess rate. T = target feature; D = distractor feature.

Do sensory distractors truly elicit multifaceted distraction effects, or do seemingly divergent outcomes stem from a unified interference mechanism? In an attempt to reconcile those seemingly divergent distraction effects, we propose the *signal-intrusion hypothesis* and have implemented the idea in a novel signal-intrusion extension of the *target-confusability-competition* framework (TCC-intrusion). Given overlaps in neural codes for visual perception and visual working memories (Harrison & Tong, 2009; LaRocque et al., 2013, 2014; Lewis-Peacock et al., 2012; Rose et al., 2016; Serences et al., 2009; Wolff et al., 2017), we propose that the perception of visual distractors can trigger an intrusion of the distractor signal into the memory-signal space. This intrusion leads to the intermingling (e.g., averaging) of the target signal with the distractor signal (Fig. 2; for analogous concepts, see Dubé et al., 2014; Fukuda et al., 2022; Zhang & Lewis-Peacock, 2024). The fundamental mechanism of signal intrusion and intermingling parallels the reconsolidation process observed in long-term memory. Actively maintaining memories renders them susceptible to intermingling with the processing of new perceptual experiences (Lee et al., 2017; Walker et al., 2003).

With this single intrusion mechanism, disparate findings in memory bias, precision, and guess rates can be reconciled by adjusting the target-distractor distance (see Fig. 2). For distractors that are highly similar to the target information, the intruded distractor signal largely overlaps with the target signal, resulting in an apparent enhancement of the target signal (manifesting as increased memory precision) as well as a shift of the memory signal toward the distractor. As the target-distractor distance increases, the same intrusion mechanism can lead to decreased memory precision. Further increases in the target-distractor distance heighten the tail of the signal through the intrusion mechanism, fostering more guesslike responses. Conceptually, the signal-intrusion mechanism appears to reconcile inconsistencies in distraction effects present in the literature.

To empirically test this potential mechanism of signal intrusion underlying distraction interferences, we developed TCC-intrusion (for TCC, see Schurgin et al., 2020) and tested the model following the rules for developing computational models proposed by Wilson and Collins (2019). We conducted data simulations to assess the reliability of recovering model parameters and to examine potential systematic trade-offs between parameters. Our model was then compared against other candidate models, including the mixture model with a bias term, which has been frequently used to measure sensory-interference effects (Li et al., 2020; Lorenc et al., 2018; Rademaker et al., 2015; Sun et al., 2017). We fitted the model to empirical data from three data sets that involved varied distraction tasks to explore how distractor intrusions varied as a function of target-distractor distance. Finally, we conducted simulations based on TCC-intrusion to assess whether the divergent distraction effects reported in the literature could be reproduced by a unified mechanism of signal intrusion.

## Research Transparency Statement

### General disclosures

**Conflicts of interest:** The authors have no conflicts of interest to declare. **Funding:** This work was completed with support from the National Institutes of Health Grant No. R01EY028746 awarded to J. A. Lewis-Peacock. This content is solely the responsibility of the authors and does not necessarily represent the official views of the National Institutes of Health. **Artificial intelligence:** ChatGPT was used to correct typos and grammatical errors specifically. No other artificial-intelligence-assisted technologies were used in this research or the creation of this article. **Ethics:** This research was approved by the Institutional Review Board of the University of Texas at Austin.

### Study disclosures

**Preregistration:** No aspects of the study were preregistered. **Materials:** All study materials are publicly available (https://doi.org/10.17605/OSF.IO/CKMNU). **Data:** All primary data are publicly available (https://osf.io/nfe8k). **Analysis scripts:** All analysis scripts are publicly available (https://doi.org/10.17605/OSF.IO/E3PG6). **Computational reproducibility:** Because of resource constraints, the reproducibility of this manuscript was not verified by the journal’s STAR team.

## Method

**Fig. 3.**
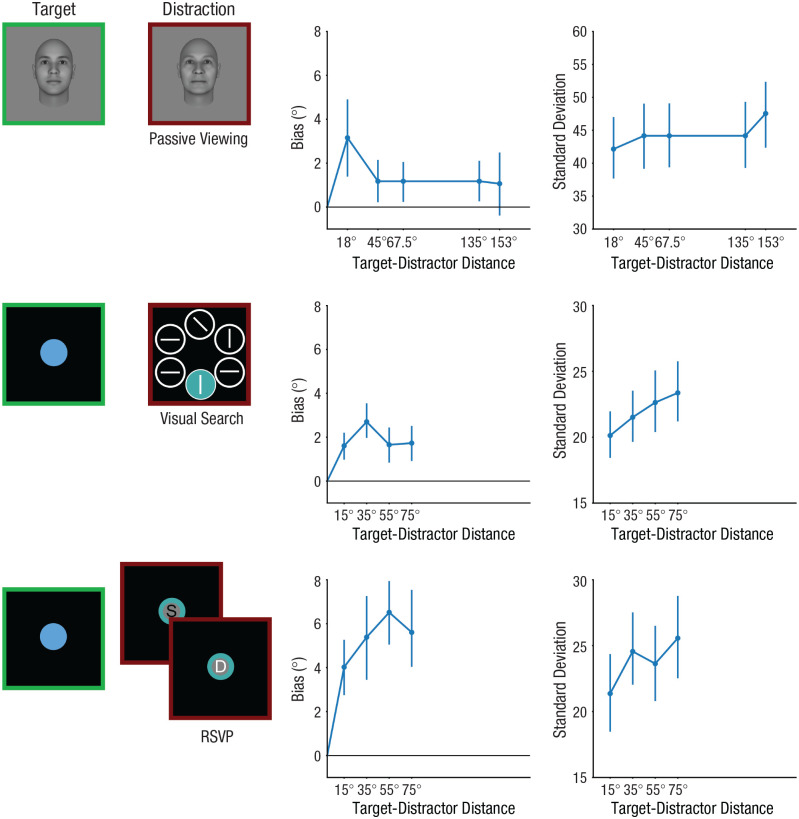
Illustrations of tasks and behavioral results from the three data sets. In all experiments, participants memorized a target feature and reproduced the memorized information after a delay. Varied distractions were inserted during the maintenance phase. In data set 1, participants memorized a target face image followed by a distracting face image. In data set 2, participants memorized a target color and completed a distracting visual-search task, reporting the direction of the only tilted bar in the search array. In data set 3, participants memorized a target color and completed a distracting rapid-serial-visual-presentation task, identifying a white-colored letter (e.g., D) among seven serially presented black letters (e.g., S). Memory biases induced by perceptual distractors followed a nonmonotonic similarity function. Biases increased initially as the target-distractor distance in the feature space (dissimilarity) increased, but then decreased as the dissimilarity increased further. Standard deviations increased as the target-distractor distance increased, indicating that distractors that were dissimilar to the target information induced greater variability in memory reports. Error bars represent 95% confidence interval.

We reanalyzed three data sets that were previously published (Mallett et al., 2020; Teng & Kravitz, 2019). The studies were approved by the institutional review board at the institution where they were originally conducted. Our data set selection is guided by two key principles: (a) ensuring a sufficient number of trials for each sampled target-distractor distance, and (b) providing adequate coverage of the full range of distances. Studies that sample the entire range of target-distractor distances typically have only a few trials per distance, posing significant challenges for modeling. To address this, we focus on data sets with fewer discrete samples of target-distractor distances but a larger number of trials for each sampled distance. We replotted memory bias and the standard deviation of error distributions to verify that the results align with the majority of prior studies (Fig. 3). Memory biases followed a nonmonotonic function associated with target-distractor distance, consistent with prior findings (Gresch et al., 2021; Nemes et al., 2011, 2012). A few studies did not observe this nonmonotonic relationship, likely because of the use of normalized bias (bias/target-distractor distance) or because of a limited range of target-distractor distances. However, by plotting raw memory bias data (Teng & Kravitz, 2019) and including additional target-distractor distances (Mallett et al., 2020), we were able to replicate the typical nonmonotonic pattern. Additionally, the standard deviation increased linearly with target-distractor distance, consistent with prior findings. Thus, the data sets selected here are representative of the broader literature.

All experiments shared similar designs, incorporating delay-estimation tasks with varied distractions during the delay period. In data set 1, a total of 120 participants were recruited through the SONA system (online psychology experiment and registration system) provided by the Department of Psychology at the University of Texas at Austin. This data set comprised four experiments, each involving 30 participants. For both data set 2 and data set 3, 50 participants were recruited for each set through Amazon Mechanical Turk.

In data set 1, memory targets were randomly chosen from a face space comprising 80 computer-generated faces that varied continuously along the dimensions of age and sex (Lorenc et al., 2014; distance between two faces = 4.5°). For data set 2 and data set 3, memory targets were selected at random from a continuous color space, encompassing 360 colors.

In all experiments, participants memorized a target feature and reproduced the memorized information after a delay with a method of adjustment response. In the experiments within data set 1, a distracting face was presented during the maintenance phase of the primary working memory task for 1 s (Fig. 3). Participants were instructed to ignore the distracting face image. For data set 2, participants completed a secondary visual-search task during the delay period. The search display comprised five white circles and one colored disk. Participants had a maximum of 4 s to locate the single tilted bar within the circles and report its orientation. In data set 3, participants completed a secondary rapid-serial-visual-presentation (RSVP) task, identifying a white-colored letter amid seven sequentially presented black letters (each lasting 0.2 s). These letters were superimposed on a colored circle. In data set 1, the target-distractor distance varied across experiments (Experiment 1: 45°; Experiment 2: 67.5°; Experiment 3: 153°; Experiment 4: 18° or 153°). In both data set 2 and data set 3, the target-distractor distance varied between trials (15°, 35°, 55°, and 75°).

The error for each trial was computed as the difference in degrees between the participant’s response and the target. To assess bias on each trial, we assigned the absolute response error a positive sign if the error was in the same direction as the distractor and a negative sign otherwise. Consequently, a positive value denotes an attractive bias, indicating a response toward the distractor, whereas a negative value denotes a repulsive bias, indicating a response away from the distractor. Signed error distributions were shown in Fig. S1 (see Supplemental Material).

### TCC-intrusion model

The TCC framework is grounded in recent evidence that error distributions from continuous reports can be characterized by a single parameter of memory strength (target *d*′), whereas the activation function of memory signals can be determined by the psychological-similarity function of the feature space (for a detailed description of the TCC, see Schurgin et al., 2020). Given that sensory distractors share neural codes with working memories (D’Esposito, 2007; D’Esposito & Postle, 2015; Harrison & Tong, 2009; Postle, 2006; Serences et al., 2009), we introduced a distractor signal in the original TCC model to represent the intruded distractor signal. The key assumption of the model is that activation functions of distractor signals should be determined by the same activation function as target signals when they are from the same feature space.

There are two parameters in our TCC signal-intrusion model. The target *d*′ is used to quantify the strength of target signals, and the distractor *d*′ is used to quantify the strength of distractor signals. Face stimuli in data set 1 lack an established psychological-similarity function; hence, we used a von Mises distribution with a parameter **κ** (concentration of the signal) to approximate activation functions of memory signals for faces. This choice was made on the basis of recent studies that demonstrated the use of a von Mises distribution to approximate memory signals, yielding comparable model fits to memory error distributions as the original psychological-similarity function (Oberauer, 2021). Therefore, the activation function for data set 1 can be described as $f\left(x\right)\;=\frac{exp\left(kcos\left(x-\theta \right)\right)}{2\pi I_{0}\left(k\right)}$ . To derive the parameter **κ**, we fitted a TCC model with only the target-signal component to the group data from a baseline condition in which no visual distractor was presented (**κ** = 1.383). For data sets 2 and 3, we used the established psychological-similarity function of the color space for constructing the activation function (see Schurgin et al., 2020).

Based on the TCC-intrusion model, on each trial, the to-be-remembered feature is boosted by target *d*′, and the signal decreases roughly exponentially as a function of distance in the feature space (Fechner’s law). The distractor feature also gets a boost by the distractor *d*′, which decreases along the distance axis, as do the target signals. Target signals are intermingled with distractor signals, and the combined signal guides memory reports. Formally, the TCC-intrusion model can be described by the following equations. Let *f*(*x*) be the activation function in the feature space:

(1) $$T(x_{1})\;=d_{T}^{\prime }\;f(x_{1})$$

(2) $$D(x_{2})\;=d_{D}^{\prime }f(x_{2})$$

(3) $$S=T(x_{1})+D(x_{2})$$

(4) r=argmax(S+ε),withε~N(0,1)

Here *x (x_1_ or x_2_)* is the potential feature value of the activation functions *f*(*x*). These functions are multiplied by signal strength *d*′. *d_T_* ′ is the strength of target activation, and *d_D_*′ is the strength of distractor activation. *T* represents the target-activation function, and *D* represents the distractor-activation function. The final activation function *S* represents the sum of target and distractor activations. Noise was drawn from a standard normal distribution and added to the activation function; then the activation function was transformed into a probability distribution via a signal-detection rule. That is, in a given trial, the feature that has the strongest activation will be selected as the final response *r*.

### Parameter recovery

Data simulations were conducted using MemToolbox (Suchow et al., 2013) and custom MATLAB scripts (The MathWorks, Natick, MA). The established psychological-scaling function of the color space (Schurgin et al., 2020) was employed to construct memory-activation and distractor-activation functions (for parameter recovery with the activation function of face stimuli, see Zhang & Lewis-Peacock, 2022). Both *d_T_*′ and distractor *d_D_*′ were randomly sampled between 0 and 1.5 with a step of 0.1 for each simulation. For each target-distractor distance between 10° and 180° with a step of 10°, we randomly sampled *d*′ 1,000 times. In each of the 1,000 simulations, data for 1,000 trials were generated. Subsequently, the simulated data were fitted to a TCC-intrusion model to estimate *d_T_*′ and *d_D_*′ values. Biases in parameter recovery were computed as the differences between recovered parameters and true parameters.

### Model comparison

The TCC-intrusion model posits that sensory interference is driven by intrusions of distractor signals, whereas the classical mixture model with a bias term suggests that sensory interference is mainly driven by shifts in memory signals (biases toward distractors). The standard mixture model with a bias term comprises two components: one for target reports, and one for guess responses. Target reports are modeled by a von Mises distribution centered at the target feature (0° in biases), whereas guesses follow a uniform distribution across the feature space (for a detailed explanation of the standard mixture model, see Zhang & Luck, 2008). Additionally, we allowed the component of target reports to shift, with a maximum shift of 15°, to capture subtle biases in target responses. The mixture model with a bias term thus has three key parameters: bias, standard deviation (inverse of memory precision), and guess rates.

To control for other major differences between models, such as decision rules (for detailed descriptions, see Oberauer, 2021), we implemented the idea of memory bias in a TCC model framework. We created a TCC-bias model in which we allowed the memory signal itself to shift.

To compare the TCC-intrusion model with other candidate models, we fitted three models to data from three data sets. We evaluated relative model fit through the Bayesian information criterion (BIC; Gelfand & Dey, 1994), which combines likelihood with a penalty for the number of free parameters.

### Skewness measurement

The skewness of individual bias distributions was calculated using the skewness function in MATLAB. Simulations were performed to assess whether the monotonic increase in skewness as a function of target-distractor distance, observed in the data sets, could be reproduced by different candidate models. Model parameters (*d_T_*′ and *d_D_*′) were first estimated through model fitting to the three data set. Based on estimated parameters, we then simulated data of 100 trials for each iteration to match the number of trials per individual in the data set. The skewness of the simulated bias distributions was then measured. The simulation process repeated 1,000 times to ensure robustness.

### Model fitting

The TCC signal-intrusion model was fitted to three data sets to derive estimations of the two parameters, *d_T_*′ and *d_D_*′. For each model fit, we used the 15,000 postconvergence samples to calculate the 95% credible interval. The 95% credible interval indicates that the true parameter value has a 95% probability of being within this interval. To compare posteriors between conditions, we computed differences between posterior samples and then calculated the 95% credible interval. Credible differences between conditions are found when the 95% credible interval of the difference posterior does not overlap with zero.

Overestimation of distractor *d*′ is more likely to occur in conditions with high target-distractor similarity compared with those with low similarity. To address such biases, we performed a control analysis. We fixed the target-distractor distance (e.g., 18°) in the model and fitted it to conditions in which the target-distractor distance varied (e.g., 18°, 45°, 135°).

If true distraction intrusions were present rather than overestimations, the estimated *d_D_*′ should be higher when distractors were indeed 18° away from the target, as opposed to when they were at distances of 45° or 135° from the target. We established a baseline for distractor *d*′ by calculating the mean of the estimated values when the distractors were not 18° away from the target. Subsequently, we subtracted this baseline from the estimated distractor *d_D_*′ when the distractors were indeed 18° away from the target. This process was repeated for each target-distractor distance. The purpose of this control analysis was to mitigate potential overestimations of distractor *d_D_*′, particularly when the target-distractor distance was close.

### Simulating model predictions

We simulated model predictions to test whether divergent distraction effects observed in the literature can be reproduced by the model. *d_T_*′ was held constant at 1, while both *d_D_*′ and target-distractor distance were systematically varied to simulate their effects on the error distribution. Exponential decay function was used to simulate the decay of *d_D_*′ with increasing target-distractor distance (see Fig. 7a), which can be described by y=exp(r*x) , where r denotes the decay rate (−0.02). Here x is the target-distractor distance, and *y* is the *d_D_*′. Simulations were conducted for 15,000 trials at each target-distractor distance between 10° and 180° with a step of 10°.

We then applied the standard mixture model with a bias term to the simulated data with an attempt to reproduce prior findings of divergent distractor effects on bias, memory precision, and guess rate.

## Results

### The TCC-intrusion model demonstrated reliable recovery of simulated parameters

**Fig. 4.**
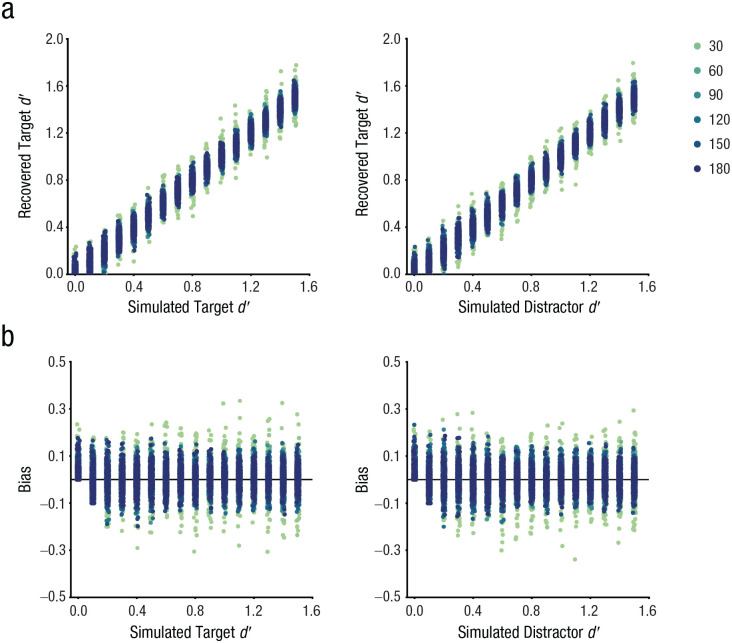
Simulated parameters successfully recovered without biases. (a) Recovered parameters showed a significant positive correlation with simulated parameters across target-distractor distances. (b) No systematic biases were observed in the recovered parameters. Legends indicate the target-distractor distance.

Both the recovered target *d_T_*′ values and *d_D_*′ values exhibited a significant positive correlation with simulated values (Fig. 4; *d_T_*′, *r =* .99, *p* < .001; *d_D_*′, *r* = .99, *p* < .001), indicating that the model reliably recovered simulated parameters. Moreover, no significant correlation was observed between recovered parameters, suggesting there were no systematic trade-offs between parameters in the model, *r =* .01, *p* = .380.

### Competitive model fitting

**Fig. 5.**
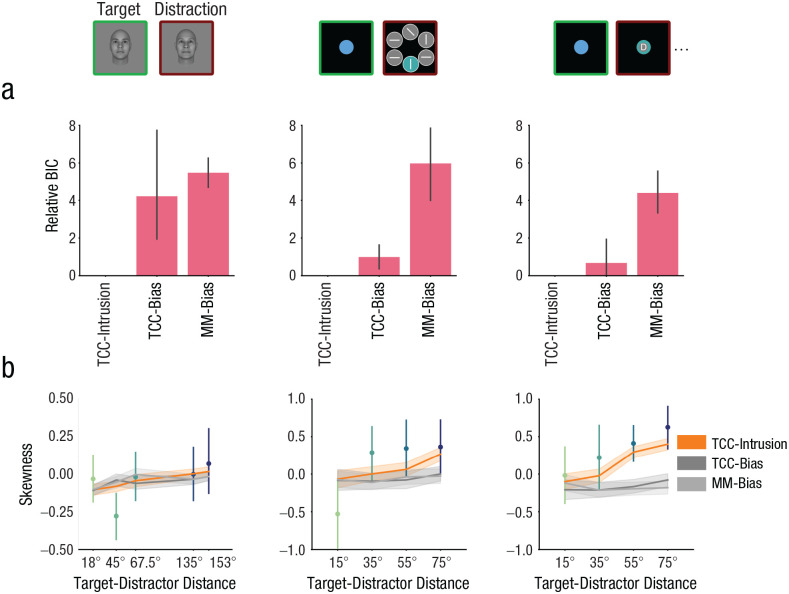
The TCC-intrusion model outperformed other candidate models in fitting the empirical data across data sets and distractor conditions. In (a) are illustrated averaged Bayesian information criteria (BICs) relative to the TCC-intrusion model. *MM* stands for mixture model; *bias* indicates a model allowing for target-signal shifts but not distractor-signal intrusion; *intrusion* indicates a model allowing for distractor-signal intrusion but not target-signal shifts. A score of zero denotes the best model. Following Raftery (1995), relative BICs of 0 to 2 indicate weak evidence in favor of the model with the smaller BIC, BICs between 2 and 6 indicate moderate evidence, and BICs between 6 and 10 indicate strong evidence. Only the TCC-intrusion model (b) successfully simulated the positive skewness in the empirical error distribution. The point plots represent the mean skewness of individual error distributions, along with error bars representing the 95% confidence intervals. The line plots illustrate the skewness of simulated error distributions based on the TCC-intrusion model, the mixture model with a bias term, and the TCC-bias model. The shaded band of the line plots represent 95% confidence intervals.

Deviations from the TCC-intrusion model in BICs were plotted in Fig. 5a. A score of zero denotes the best model. Both the TCC-intrusion model and the TCC bias model outperformed the mixture model in fitting error distributions across data sets and target-distractor distances. Critically, the TCC-intrusion model had better model fits compared with both bias models in the mixture-model framework and the TCC framework. This suggests that incorporating a distractor-intrusion mechanism in a model likely provides a more accurate description of empirical data than models incorporating a memory-signal-shift mechanism.

The integration of a distractor signal with a target signal leads to the memory signal being skewed in the direction of the distractor, resulting in positive skewness in the bias distributions. This intrusion-driven skewness should increase as the distractor becomes more dissimilar from the target information (Fig. 2). Indeed, the skewness of the bias distributions, measured for each individual subject, showed a monotonic increase as the target-distractor distance increased (Fig. 5b). Notably, only the TCC-intrusion model was able to account for this positive skewness, whereas models that implemented a memory-signal-shift mechanism failed to capture this change in skewness.

#### The TCC-intrusion model revealed that distractor interference effects diminished with an increase in target-distractor distance

**Fig. 6.**
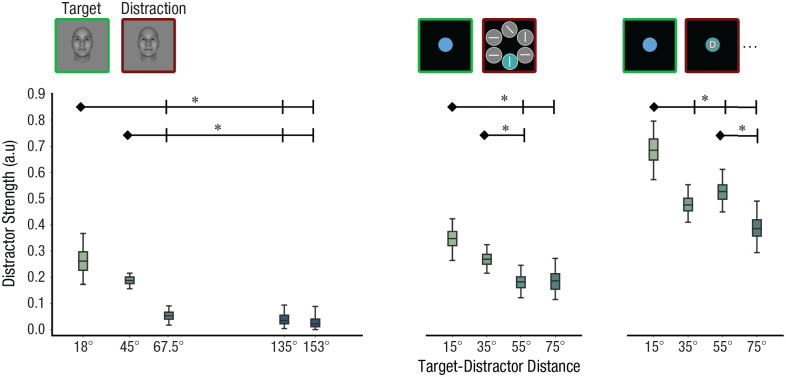
Consistent decrease in distractor *d_D_*′ with increasing target-distractor distance across data sets. Results from data set 1 (face stimuli with passively viewed distractions), 2 (color stimuli with visual search distraction tasks), and 3 (color stimuli with RSVP distraction tasks) are presented from left to right respectively. Box plots depict the 95% posterior distributions of distractor *d_D_*′ values obtained from model fitting. Error bars represent 95% credible intervals. *d_D_*′ exhibits a decay pattern as the target-distractor distance increases. The asterisk denotes that the credible intervals for the differences between the two conditions do not include 0. a.u. = arbitrary units.

We fitted the TCC-intrusion model to group data from three data sets with varied target-distractor distances and diverse distraction tasks. *d_D_*′ exhibited a notable decrease as the target-distractor distance increased (Fig. 6). In data set 1, for instance, the *d_D_*′ decreased substantially as the target-distractor distance increased from 45° to 67.5° (18° vs. 67.5°: $\hat{\theta }$ = 0.210, difference CI (credible interval) = [0.115, 0.316]; 18° vs. 135°: $\hat{\theta }$ = 0.223, difference CI = [0.119, 0.333]; 18° vs. 153°: $\hat{\theta }$ = 0.231, difference CI = [0.125, 0.340]; 45° vs. 67.5°: $\hat{\theta }$ = 0.134, difference CI = [0.086, 0.184]; 45° vs. 135°: $\hat{\theta }$ = 0.147, difference CI = [0.086, 0.184]; 45° vs. 153°: $\hat{\theta }$ = 0.155, difference CI = [0.081, 0.204]). Likewise, in data sets 2 and 3, there was a consistent decrease in *d_D_*′ as the target-distractor distance increased (data set 2, 15° vs. 55°: $\hat{\theta }$ = 0.160, difference CI = [0.066, 0.257]; 15° vs. 75°: $\hat{\theta }$ = 0.157, difference CI = [0.048, 0.257]; 35° vs. 55°: $\hat{\theta }$ = 0.084, difference CI = [0.009, 0.160]; data set 3, 15° vs. 35°: $\hat{\theta }$ = 0.206, difference CI = [0.074, 0.337]; 15° vs. 55°: $\hat{\theta }$ = 0.158, difference CI = [0.020, 0.290]; 15° vs. 75°: $\hat{\theta }$ = 0.295, difference CI = [0.139, 0.436]; 55° vs. 75°: $\hat{\theta }$ = 0.138, difference CI = [0.007, 0.265]).

As distractors become similar to the target information, the model faces challenges in distinguishing between target and distractor activation functions from the error distribution. This complexity may lead to the overestimation of *d_D_*′ values when the target-distractor distance is close. To address this concern, we conducted a control analysis. We computed the empirical baseline of *d_D_*′ by fixing the target-distractor distance (e.g., 18°) in the model and fitting it to conditions in which the target-distractor distance deviated from the model assumption (e.g., 45°, 67.5°, 135°, 153°). This baseline was subtracted from estimated *d_D_*′ in the condition in which distractors were indeed 18° away from the target. We repeated this control analysis for each of the target-distractor distances. We successfully replicated the decrease in *d_D_*′ with increasing target-distractor distance in this control analysis, suggesting that this pattern is not a result of biases in the model (data set 1, 18° vs. 67.5°: $\hat{\theta }$ = 0.172, difference CI = [0.052, 0.302]; 18° vs. 135°: $\hat{\theta }$ = 0.211, difference CI = [0.081, 0.355]; 18° vs. 153°: $\hat{\theta }$ = 0.184, difference CI = [0.056, 0.315]; 45° vs. 67.5°: $\hat{\theta }$ = 0.125, difference CI = [0.061, 0.191]; 45° vs. 135°: $\hat{\theta }$ = 0.164, difference CI = [0.079, 0.263]; 45° vs. 153°: $\hat{\theta }$ = 0.137, difference CI = [0.056, 0.207]; data set 2, 15° vs. 55°: $\hat{\theta }$ = 0.175, difference CI = [0.021, 0.308]; 15° vs. 75°: $\hat{\theta }$ = 0.240, difference CI = [0.078, 0.403]; 35° vs. 55°: $\hat{\theta }$ = 0.138, difference CI = [0.021, 0.258]; 35° vs. 75°: $\hat{\theta }$ = 0.203, difference CI = [0.070, 0.336]; data set 3, 15° vs. 75°: $\hat{\theta }$ = 0.267, difference CI = [0.049, 0.486]; 35° vs 75°: $\hat{\theta }$ = 0.192, difference CI = [0.013, 0.369]).

#### The TCC-intrusion model proposes a unified mechanism underlying diverse sensory-interference effects

**Fig. 7.**
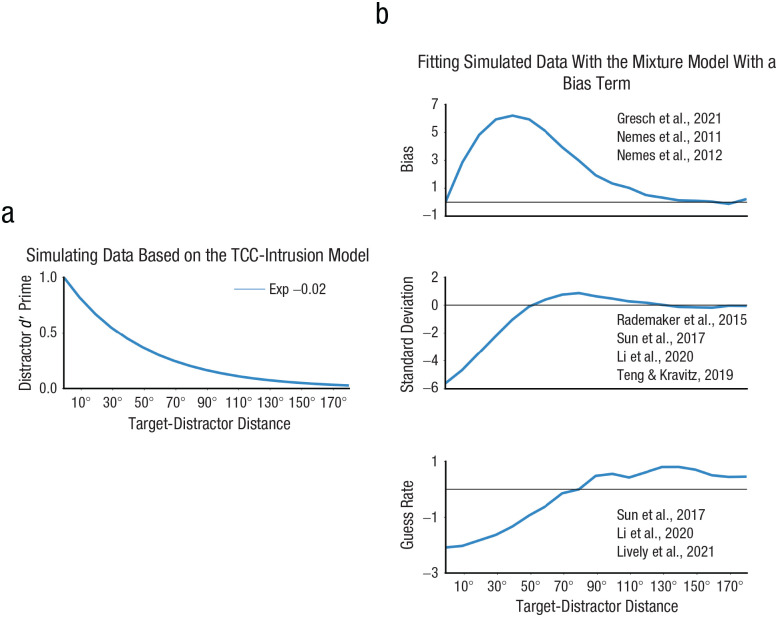
Simulations from the TCC-intrusion model replicated varied distraction effects on bias, standard deviations (1/precision), and guess rates. In data simulations (a), the distractor *d_D_*′ decreased as a function of target-distractor distance. Here we simulated the decay as a exponential function (Exp) with a decay rate of –0.02. In (b), from the top to the bottom row are shown relative changes in bias, standard deviation, and guess rate, compared with the baseline condition, as revealed by fitting the mixture model with a bias term to the simulated data. References were included to indicate prior studies that showed similar results. TCC-intrusion = target-confusability-competition intrusion.

Can our model account for the divergent findings of sensory-interference effects in the literature? To address this, we simulated data on the basis of our TCC-intrusion model and fitted the mixture model with a bias term to replicate prior findings. We compared estimated parameters for 18 target-distractor distances (0°–180°) to a no-distraction condition. Differences in estimated parameters between distraction conditions and the no-distraction condition were plotted (see Fig. 7). Simulated biases showed an inverted U-shape as a function of target-distractor distance, consistent with prior reports (Gresch et al., 2021; Nemes et al., 2011, 2012). As the target-distractor distance increased, memory biases initially increased and then returned to 0. In standard deviations (inverse of memory precision), distractors that were similar to the target information led to increased memory precision (10°–40°), intermediately similar distractors led to decreased precision (40°–100°), whereas dissimilar distractors caused no significant changes in precision (100°–180°). This is consistent with prior reports that similar distractors tended to increase memory precision whereas intermediately similar distractors tended to decrease memory precision (Li et al., 2020; Rademaker et al., 2015; Sun et al., 2017). Finally, in guess rates, replicating prior research (Li et al., 2020; Sun et al., 2017), similar and intermediately similar distractors led to reduced guess rates (10°–80°), whereas dissimilar distractors led to increased guess rates (90°–180°). From simulating model predictions, we were able to replicate prior divergent findings of sensory-interference effects in biases, memory precision, and guess rates. This suggests that the seemingly disparate results regarding sensory-interference effects in the literature can be explained through a unified mechanism of distractor-signal intrusion.

## Discussion

How do perceptual distractors interfere with working memories? Prior studies based on mixture modeling suggested that there might be multiple mechanisms underlying distraction effects, but we have proposed that the perception of visual distractors results in a singular effect—namely, the intrusion of distractor signals into the memory-signal space. Consequently, the memory signal for the target becomes intermingled with the intruded distractor signal and influences memory responses. We thoroughly tested this model through various analyses of three existing data sets, including parameter recovery, model comparison, fitting, and prediction simulations. Our results indicated that the TCC-intrusion model reliably recovered simulated parameters and exhibited superior fits to empirical data compared with prominent alternative models. The model revealed that the interference of perceptual distractors diminished as the target-distractor distance increased, aligning with predictions from the sensory-recruitment hypothesis. Importantly, simulated predictions based on the TCC-intrusion model successfully replicated prior diverse findings related to sensory-interference effects on response bias, memory precision, and guess rate. This suggests that signal intrusion may serve as a unified mechanism underlying the seemingly disparate perceptual-interference effects observed in the literature.

The idea of signal intrusion helps to resolve key debates surrounding sensory interference in the literature. The traditional viewpoint has held that sensory interference results from shifts in target memory signals toward distractors (Hallenbeck et al., 2021; Lorenc et al., 2018; Mallett et al., 2020; Rademaker et al., 2015; Sun et al., 2017; Teng & Kravitz, 2019). However, this memory-shift hypothesis is inconsistent with recent evidence showing distractor effects on memory precision and guess rates (Li et al., 2020; Rademaker et al., 2015; Sun et al., 2017). Intriguingly, some studies even suggest that introducing perceptual distractors can enhance memory precision and reduce guess rates, challenging the notion that perceptual distractors universally interfere with memories. These divergent findings imply that the impact of perceptual distractors on working memory is complex and may involve multiple mechanisms. In contrast, our signal-intrusion hypothesis provides a unified explanation for these divergent findings. By varying the target-distractor distance, the same signal-intrusion mechanisms can account for changes in memory bias, memory precision, and guess rates. This offers a comprehensive framework to interpret the seemingly multifaceted effects of perceptual distractors on working memory performance.

Signal intrusion potentially occurs through the overlap in representations of visual perception and visual working memories (D’Esposito, 2007; D’Esposito & Postle, 2015; Harrison & Tong, 2009; Pasternak & Greenlee, 2005; Postle, 2006, 2015; Serences et al., 2009). Accumulating evidence has shown that machine-learning algorithms trained to differentiate neural patterns associated with visual perception can successfully differentiate between neural patterns associated with maintaining visual working memories in the early visual cortices, suggesting that visual perception and visual working memory have shared coding formats (Harrison & Tong, 2009; LaRocque et al., 2013, 2014; Lewis-Peacock et al., 2012; Rose et al., 2016; Serences et al., 2009; Wolff et al., 2017). This shared coding format implies that the sensory representations generated during the visual perception of distractors can closely resemble those involved in maintaining visual working memories. The merging of these two signals, due to their shared neural representation, can disrupt the original memory signal and ultimately lead to interference in the maintenance and/or retrieval of target information from working memory.

In delayed-estimation tasks, previous studies have consistently revealed a nonmonotonic relationship between memory biases and target-distractor similarity. This finding stands in direct contrast to both the sensory-recruitment hypothesis and the congruence effect observed in recognition paradigms. Our model-fitting results suggest the existence of a parallel congruency effect in the feature space, reconciling prior discrepancy between recognition and estimation paradigms. Our findings align with predictions of the sensory-recruitment hypothesis of working memory (D’Esposito & Postle, 2015; Scimeca, et al., 2018), demonstrating that the intrusion of distractor signals decreases as the similarity between target and distractor decreases. This pattern is also consistent with the congruency effect observed in previous research in which distractors from the same category as the target information tend to impair working memory performance more than distractors from different categories (Bloemendaal et al., 2015; Hermann et al., 2021; Jha et al, 2004; Jha & Kiyonaga, 2010; Yoon et al., 2006). Collectively, these findings converge to suggest that the impact of perceptual distractors on working memories follows a similarity function. Specifically, the impact of the memory signal by distractors increases as the similarity between the target and distractor information increases.

The finding that perceptual interference follows a similarity function adds to our understanding of bidirectional interactions between visual perception and visual working memory. It is well established that maintaining information in working memory can influence the processing of external information, resulting in enhanced and prioritized processing of matching information in the environment (Carlisle et al., 2011; Kang et al., 2011; Kiyonaga & Egner, 2013; Olivers et al., 2011; Soto et al., 2008; Teng & Kravitz, 2019). Our work, along with previous research, complements this narrative by demonstrating the reciprocal relationship. Perceiving information from the environment also influences working memory representations, with a more pronounced effect when there is a greater match between the representations of perception and working memory. This bidirectional interaction underscores the intricate interplay between these cognitive processes.

The implementation of the TCC-intrusion model has several practical limitations that should be addressed in future research. The model was specifically designed to elucidate attraction biases induced by perceptual distractors on working memory responses. However, examination of individual data from the current study and others reveals that some individuals may exhibit a general repulsive or negative bias in their responses, directly contradicting the assumptions of the TCC-intrusion model and making it less adept at capturing such instances. It remains unclear how and why those repulsive biases might occur. Repulsive biases were typically found between items already in working memory (Bae & Luck 2017; Chunharas et al., 2022) but were rarely found at the group level between working memory contents and perceptual distractors. It could be that some participants developed the strategy of choosing to focus on features that were further away from the perceptual distractors, leading to repulsive biases, or it could be that their memory representations were indeed biased away from perceptual distractors. Future research is needed to look into these individual differences. In the current model, we used the von Mises distribution to model memory activation functions. The von Mises distribution is commonly applied to circular data, similar to what we used here—such as orientation, color, location, and artificial faces (van den Berg et al., 2012; Zhang & Luck, 2008). However, it is not guaranteed to work for all arbitrary stimulus spaces. Future studies should examine the choice of activation functions for modeling error distributions. We used the TCC model with uncorrelated noise in this study. Although this version of the TCC model has shown a superior fit to empirical data in previous work (Oberauer, 2021), the original TCC model used correlated noise, which was estimated from independent perceptual-matching tasks. Future research could explore whether the use of correlated versus uncorrelated noise affects model performance. The data were primarily collected from college students, which may limit the generalizability of our findings.

Finally, we would like to provide some practical guidelines for measuring sensory interference, aiming to address the potential for divergent conclusions resulting from different measurements of the sensory-interference effect. Considering the underlying mechanisms of sensory interference is crucial when selecting measurements. From both theoretical and statistical perspectives, we have demonstrated the superiority of signal intrusion as a candidate mechanism underlying sensory interference compared with memory-signal shifts. Therefore, for computational models, we recommend utilizing our TCC-intrusion model or similar signal-average models (Dubé et al., 2014; Fukuda et al., 2022) over the classical mixture model with a bias term. For nonmodeling measurements, we propose using normalized biases (bias/target-distractor distance) over raw biases and absolute errors. There are two primary reasons for this recommendation. First, the mechanism of signal intrusion does not invariably result in the impairment of working memory performance, particularly when distractors closely resemble the target (Fig. 7). Consequently, measuring how memory performance is impaired through absolute errors may not accurately capture perceptual interferences in delayed-estimation tasks. Second, the mechanism of signal intrusion implies that raw biases are influenced by both the strength of distractor intrusion and the target-distractor distance. With the same distractor-intrusion strength, increasing the target-distractor distance alone can lead to more responses further away from the target locations, consequently inflating raw biases. To isolate the effect of the strength of distractor intrusion, we recommend normalizing raw biases on the basis of target-distractor distance.

In conclusion, we proposed and tested a novel signal-intrusion extension of the TCC model to account for divergent distracting effects through a unified mechanism. Our findings indicate that the influence of visual distractors on working memory diminishes as the target-distractor similarity decreases, in accordance with predictions by the sensory-recruitment hypothesis. Our results suggest that distraction interference is likely driven by the mechanism of signal intrusion rather than by memory-signal shifts.

## Supplemental Material

sj-docx-1-pss-10.1177_09567976251331039 – Supplemental material for Signal Intrusion Explains Divergent Effects of Visual Distraction on Working Memory Supplemental material, sj-docx-1-pss-10.1177_09567976251331039 for Signal Intrusion Explains Divergent Effects of Visual Distraction on Working Memory by Ziyao Zhang and Jarrod A. Lewis-Peacock in Psychological Science

## Transparency

*Action Editor*: Vishnu Sreekumar

*Editor*: Simine Vazire

*Author Contributions*

**Ziyao Zhang:** Conceptualization, Formal analysis, Investigation, Methodology, Software, Visualization, Writing – original draft, Writing – review & editing.

**Jarrod A. Lewis-Peacock:** Funding acquisition, Supervision, Writing – review & editing.

**Artificial Intelligence:** ChatGPT was used to correct typos and grammatical errors specifically. No other artificial-intelligence-assisted technologies were used in this research or the creation of this article.

**Ethics:** This research was approved by the Institutional Review Board of the University of Texas at Austin.

**Open Practices:** Study disclosures. Preregistration: No aspects of the study were preregistered. Materials: All study materials are publicly available (https://doi.org/10.17605/OSF.IO/CKMNU). Data: All primary data are publicly available (https://osf.io/nfe8k). Analysis scripts: All analysis scripts are publicly available (https://doi.org/10.17605/OSF.IO/E3PG6). Computational reproducibility: Because of resource constraints, the reproducibility of this manuscript was not verified by the journal’s STAR team.
