# Supplementary material for: Signal Intrusion Explains Divergent Effects of Visual Distraction on Working Memory
Source: Psychol Sci. 2025 May 5;36(5):316–31. doi: 10.1177/09567976251331039 (PMC12369972; doi:10.1177/09567976251331039)
Supplement: sj-docx-1-pss-10.1177_09567976251331039 – Supplemental material for Signal Intrusion Explains Divergent Effects of Visual Distraction on Working Memory [file sj-docx-1-pss-10.1177_09567976251331039.docx]

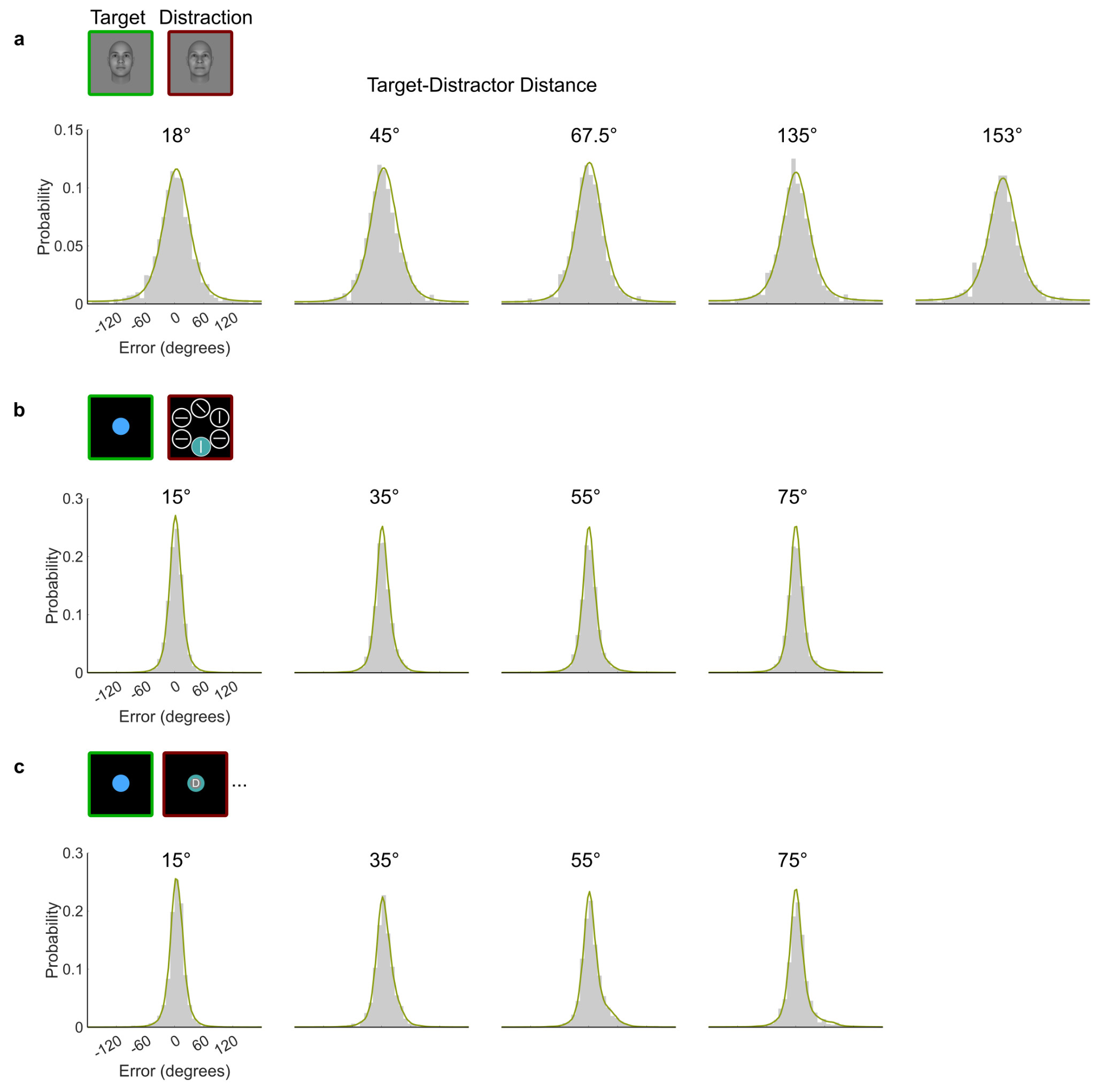


S Fig 1. **Bias distributions and model fittings across conditions and datasets.** Gray hist plots represent bias distributions across participants. Green lines represent model predictions based on fitted parameters (maximum a posteriori, MAP).
